# Microenvironmental Control of Thyroid Cancer Plasticity and Radioiodine Resistance

**DOI:** 10.3390/ijms27167305

**Published:** 2026-08-16

**Authors:** Qing Ye, Xiao-Ping Yu

**Affiliations:** Zhejiang Provincial Key Laboratory of Biometrology and Inspection and Quarantine, College of Life Science, China Jiliang University, Hangzhou 310018, China; yeq202511@163.com

**Keywords:** thyroid cancer, dedifferentiation, tumor microenvironment, cancer-associated fibroblasts, tumor-associated macrophages, cellular plasticity, lymphatic metastasis, radioiodine resistance, extracellular vesicles

## Abstract

Follicular-cell-derived thyroid cancers that progress from differentiated tumors to poorly differentiated or anaplastic states commonly lose thyroid lineage identity, radioiodine avidity, and favorable clinical behavior. Genetic and signaling alterations within tumor cells explain part of this transition, but they do not fully account for the coexistence of different differentiation states within the same molecular subtype or even within the same lesion. Increasing functional evidence indicates that dedifferentiation is maintained by reciprocal interactions between malignant cells and the immune, stromal, metabolic, and inflammatory microenvironment. Cancer-associated fibroblast glycolysis and lactate release, IL-6/CXCL8-driven inflammatory signaling, hypoxia, transforming growth factor-beta signaling, and tumor-associated macrophage feedback can suppress thyroid lineage programs while promoting plasticity, invasion, and treatment resistance. Here, we synthesize mechanistic studies supported by genetic perturbation, co-culture, pharmacologic blockade, iodine-uptake assays, animal models, or patient-level radioiodine endpoints. We distinguish functional dedifferentiation from epithelial–mesenchymal transition, stemness, and lymph-node metastasis, and discuss therapeutic strategies that combine tumor-cell redifferentiation with targeting of microenvironmental feedback to restore durable radioiodine sensitivity.

## 1. Introduction

Thyroid cancer is the most common endocrine malignancy and shows marked clinical heterogeneity. Most follicular-cell-derived tumors are differentiated thyroid cancers (DTCs), mainly papillary thyroid carcinoma (PTC) and follicular thyroid carcinoma. These tumors usually retain thyroid-lineage features and iodine-handling capacity. Outcomes are favorable after surgery, thyroid-stimulating hormone suppression, and radioactive iodine (RAI) therapy. A subset of DTCs, however, develops local recurrence, distant metastasis, or RAI resistance, and a smaller fraction progresses to poorly differentiated thyroid carcinoma (PDTC) or anaplastic thyroid carcinoma (ATC), where treatment options narrow and survival deteriorates [1,2].

Dedifferentiation refers to the progressive loss of follicular-cell lineage identity and thyroid-specific functions. It includes reduced expression of PAX8, NKX2-1, FOXE1, thyroglobulin (TG), thyroid peroxidase (TPO), thyroid-stimulating hormone receptor (TSHR), and the sodium–iodide symporter (NIS/SLC5A5), leading to impaired iodine uptake and thyroid hormone biosynthesis [3,4,5,6]. Genomic studies have established BRAF- and RAS-driven MAPK signaling as a major axis of PTC molecular subtyping and differentiation status [3]. During progression toward PDTC and ATC, alterations involving the TERT promoter, TP53, PI3K-AKT signaling, chromatin remodeling, and cell-cycle control accumulate [7,8,9,10,11,12,13]. Clinical cohorts further show that lower thyroid differentiation scores are associated with shorter progression-free and overall survival, whereas metastatic tumors with lower MAPK output and higher differentiation scores are more likely to show structural responses to RAI [11,12,13,14,15,16]. Importantly, dedifferentiation, epithelial–mesenchymal transition (EMT), stemness, and lymph-node metastasis overlap mechanistically but are not interchangeable concepts. In PTC, nodal metastasis can occur while thyroid-lineage function is still largely preserved.

A tumor-cell-centric genetic model is therefore incomplete. It cannot readily explain why tumors sharing a driver genotype may differ in differentiation state, or why differentiated and anaplastic regions may coexist within the same tumor. Recent single-cell and spatial studies suggest that progression from DTC to ATC involves malignant-state transitions, macrophage immunosuppression, fibroblast expansion, and T-cell dysfunction [17,18,19,20,21,22,23,24,25]. These omics studies are most useful as maps of candidate states and interactions; causal inference still requires functional testing. Such evidence is emerging. Hypoxia and endoplasmic reticulum stress can induce IL-6 and CXCL8, suppress iodine-metabolism genes, and reduce RAI uptake [26]. Loss of PDCD4 in tumor cells promotes eIF4A-dependent translation of immunosuppressive factors and enrichment of thymidine phosphorylase (TYMP)-positive macrophages [27]. Cancer-associated fibroblasts (CAFs) can drive dedifferentiation through a ZFP57-PKM2-lactate-TGF-β/Smad2/3 axis that reduces iodine uptake [28]. Macrophage-derived CXCL16 can enhance PTC-cell migration through CXCR6-AKT signaling while reinforcing immunosuppressive macrophage phenotypes [29].

**Figure 1:**
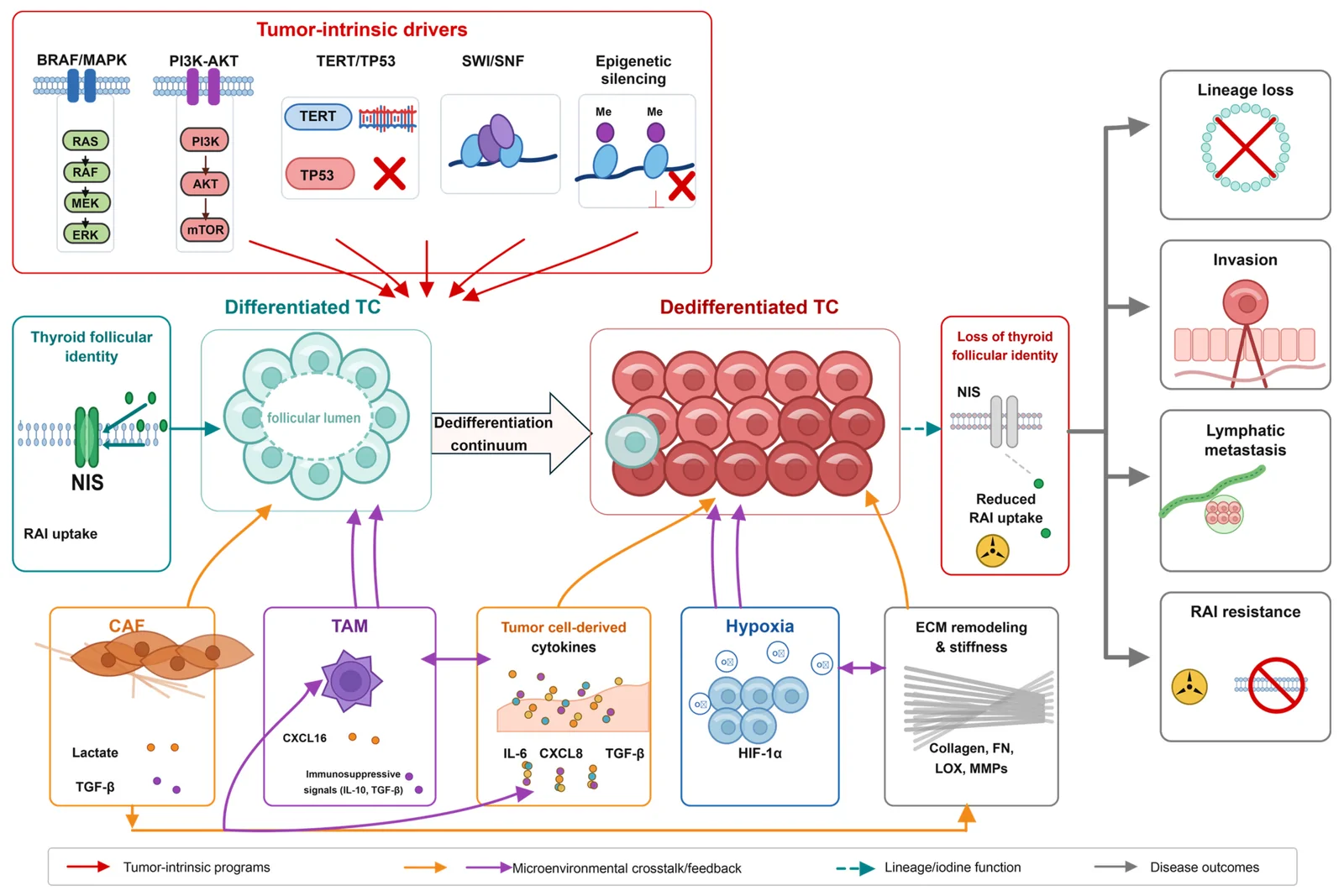
Bidirectional tumor–microenvironment model of thyroid cancer dedifferentiation. Tumor-intrinsic drivers, including BRAF/MAPK, PI3K-AKT, TERT/TP53, SWI/SNF disruption, and epigenetic silencing, converge on a dedifferentiation continuum. Loss of thyroid follicular identity and impaired NIS-mediated iodine uptake are reinforced by CAFs, TAMs, tumor cell-derived cytokines, hypoxia, and ECM remodeling, resulting in lineage loss, invasion, lymphatic metastasis, and RAI resistance. Abbreviations: CAFs, cancer-associated fibroblasts; ECM, extracellular matrix; NIS, sodium–iodide symporter; RAI, radioactive iodine; TAMs, tumor-associated macrophages. This figure was created using Adobe Illustrator 2025 (Version 29). Certain biological motifs were manually redrawn and modified by the authors with reference to materials by Servier from Servier Medical Art, accessed through Bioicons. The original source materials are licensed under the Creative Commons Attribution 3.0 Unported license (CC BY 3.0; https://creativecommons.org/licenses/by/3.0/, accessed on 25 June 2026), and all modifications and final figure composition were made by the authors. Colored circles represent representative soluble mediators and are schematic; their colors do not indicate quantitative abundance.

This review frames thyroid cancer dedifferentiation as a dynamic state shaped by tumor-intrinsic programs and microenvironmental signals. We focus on how malignant cells remodel CAFs, tumor-associated macrophages (TAMs), inflammatory circuits, and extracellular-matrix components, and how these compartments feedback to stabilize lineage loss, RAI resistance, and invasive behavior. We prioritize studies with genetic perturbation, co-culture, pharmacologic blockade, iodine-uptake assays, or in vivo validation, and separate thyroid functional dedifferentiation from EMT, stemness, and lymphatic spread. This distinction is essential for evaluating redifferentiation strategies that target both malignant-cell programs and the ecosystem that maintains them. The overall bidirectional model is summarized in Figure 1.

## 2. Defining Thyroid Cancer Dedifferentiation

### 2.1. Functional and Molecular Definition of Dedifferentiation

The differentiated state of thyroid follicular cells is maintained by transcriptional programs that preserve lineage identity and thyroid hormone synthesis. PAX8, NKX2-1, and FOXE1 regulate TG, TPO, TSHR, and NIS/SLC5A5. NIS must also localize correctly to the basolateral membrane to mediate active iodide uptake. In thyroid cancer, NIS protein may remain detectable but be retained intracellularly, leaving iodide transport functionally impaired [6]. Dedifferentiation is therefore better defined by progressive disruption of thyroid-lineage regulation and iodine-handling function than by morphology alone [1,4,5,6,30,31,32].

Assessing dedifferentiation requires evidence at several levels. Loss of differentiation-related transcripts or proteins suggests lineage impairment, but it does not by itself prove functional dedifferentiation. Defective NIS membrane trafficking can impair iodine uptake even when transcription is not strongly reduced [6]. Stronger evidence combines thyroid differentiation scores, NIS protein localization, in vitro or in vivo radioiodine uptake, and lesion-level iodine avidity in patients. Experimental restoration of NIS transcription, glycosylation, and membrane transport can increase iodide uptake in ATC cells, further separating protein abundance from function [33]. Dedifferentiation spans a continuum from preserved thyroid function to near-complete lineage erasure, rather than a binary label [4,5,6,14,15].

### 2.2. Histological Progression and Clinical Consequences

Follicular-cell-derived thyroid cancers form a histological spectrum from DTC to PDTC and ATC. PTC and follicular thyroid carcinoma usually retain follicular architecture and partial thyroid-specific function. PDTC shows reduced follicular architecture, higher proliferative activity, and diminished differentiation markers, whereas ATC typically lacks thyroid-specific morphology and function. The Turin proposal defines PDTC by solid, trabecular, or insular growth, absence of PTC nuclear features, and at least one high-grade feature [34]. The fifth edition of the World Health Organization classification further separates PDTC from differentiated high-grade thyroid carcinoma, underscoring that histological differentiation and proliferative grade are related but distinct pathological dimensions [11,34,35].

Clinically, dedifferentiation is linked to aggressive behavior, RAI resistance, recurrence, and poor survival. In PTC, lower differentiation scores are associated with adverse outcomes [14]. In metastatic thyroid cancer, differentiated tumors retain a higher likelihood of RAI response, whereas less differentiated tumors are frequently RAI refractory [15,36]. Nevertheless, histology is an imperfect proxy for functional iodine handling. Some metastases retain iodine avidity despite aggressive features, whereas other lesions lose RAI uptake without overt anaplastic morphology. A useful framework must therefore integrate histology, molecular drivers, thyroid-lineage transcription, NIS localization, and functional uptake.

### 2.3. Distinguishing Dedifferentiation from EMT, Stemness, and Metastasis

Dedifferentiation often intersects with EMT, stemness, invasion, and metastatic dissemination, but these processes answer different biological questions. Dedifferentiation asks whether the tumor still behaves like thyroid follicular epithelium. EMT asks whether epithelial adhesion and polarity have been replaced by migratory programs. Stemness refers to self-renewal and therapy tolerance. Metastasis requires invasion, survival in transit, colonization, and adaptation to a secondary site [37]. The operational distinctions used in this review are summarized in Table 1.

The same pathways may contribute to several of these outputs. MAPK, PI3K-AKT, TGF-β, hypoxia, inflammatory cytokines, and mechanical signaling can suppress thyroid-lineage genes while promoting EMT or invasion [38,39,40,41,42,43]. This overlap obscures mechanisms when readouts are combined too broadly. A study that shows increased migration or EMT markers does not necessarily demonstrate thyroid functional dedifferentiation unless NIS, thyroid-lineage factors, iodine uptake, or RAI response are examined. Throughout this review, dedifferentiation is used for lineage and iodine-handling loss, while EMT, stemness, and metastasis are treated as related but non-equivalent phenotypes. In particular, lymph-node metastasis is considered a related clinical outcome of invasion and microenvironmental remodeling, rather than a surrogate marker of functional dedifferentiation or RAI resistance.

**Table 1:** Operational definitions of thyroid cancer dedifferentiation-related phenotypes.

Phenotype	Working Definition in This Review	Key Readouts	Representative References
Dedifferentiation	Progressive loss of thyroid follicular identity and thyroid-specific function.	Lower PAX8, NKX2-1, FOXE1, TG, TPO, TSHR and NIS/SLC5A5; reduced thyroid differentiation score; impaired iodine uptake.	[3,4,7,14]
Impaired iodine handling/RAI resistance	Failure to concentrate or respond to radioactive iodine, driven by reduced NIS expression, abnormal NIS localization or survival signaling.	Low RAI avidity; reduced membrane NIS; discordance between NIS expression and iodine uptake; response to redifferentiation therapy.	[4,6,44,45,46,47]
EMT and local invasion	Acquisition of motile and matrix-invasive features, which may accompany but does not equal dedifferentiation.	Reduced E-cadherin; increased vimentin, SNAIL/SLUG/ZEB1; MMP-2/MMP-9/MMP-14; Matrigel or organotypic invasion assays.	[37,42,48,49]
Stem-like plasticity	Acquisition of self-renewal, tumor-initiating or therapy-tolerant cellular states.	CD44, ALDH activity, SOX2, NANOG, OCT4; sphere/organoid formation; tumor-initiation assays.	[42,50]
Lymphatic metastatic competence	Capacity to enter lymphatic vessels and colonize regional lymph nodes; this can occur even in differentiated PTC.	Lymphovascular invasion; nodal metastasis; VEGF-C/VEGFR3, VEGF-D, LYVE1, PROX1 and podoplanin-positive lymphatics.	[51,52,53,54,55]
Microenvironmental reinforcement	Stromal, immune, hypoxic and matrix cues that stabilize low-differentiation, invasive or RAI-resistant states.	CAF/TAM infiltration; IL-6, CXCL8, TGF-β; hypoxia/HIF-1α; ECM stiffness; collagen remodeling; YAP/TAZ activation.	[21,56,57,58,59,60,61,62,63,64,65,66,67,68]

Abbreviations: EMT, epithelial–mesenchymal transition; NIS, sodium–iodide symporter; RAI, radioactive iodine; CAF, cancer-associated fibroblast; TAM, tumor-associated macrophage; ECM, extracellular matrix. These operational distinctions are summarized in Table 1.

## 3. Tumor-Intrinsic Initiators of Dedifferentiation

### 3.1. MAPK and PI3K-AKT Signaling Suppress Thyroid Lineage Identity

Dedifferentiation often begins with sustained oncogenic suppression of thyroid-lineage transcription. In PTC, BRAF, RAS, and receptor tyrosine kinase fusions activate MAPK signaling, but their signaling intensity and differentiation phenotypes differ. The Cancer Genome Atlas showed that BRAF-like tumors have stronger MAPK output and lower thyroid differentiation scores, whereas RAS-like tumors more often retain follicular structure and iodine-metabolism programs [3,13]. Molecular analyses of metastatic disease likewise link lower MAPK output and higher differentiation scores to structural responses after RAI therapy [15]. These findings suggest that dedifferentiation depends not only on the identity of the driver alteration, but also on the magnitude, duration, and feedback architecture of pathway activation.

Mouse models with inducible *BRAF^V600E^* provide direct evidence that sustained MAPK signaling can cause functional dedifferentiation. *BRAF^V600E^* activation disrupts follicular architecture, thyroid-specific gene expression, and radioiodine uptake, whereas BRAF withdrawal or RAF/MEK inhibition can partially restore these features [69]. Restoration of NIS, TG, TPO, and TSHR requires ERK activity to fall below a critical threshold [70]. However, HER3 transcription and NRG1 autocrine feedback can reactivate ERK after RAF or MEK inhibition, limiting both the magnitude and durability of redifferentiation [71]. Thus, the response is better understood as a signaling-dose effect with a feedback-dependent threshold than as a simple on-off switch.

MAPK signaling also promotes lineage loss through secondary signaling circuits. *BRAF^V600E^* can induce TGF-β secretion and autocrine TGF-β/Smad activity, suppress NIS, and increase invasive behavior; blocking this circuit partially restores NIS and reduces EMT-like changes [38]. MED16 downregulation and REG-γ-mediated degradation of Smad7 can further enhance TGF-β signaling, reduce thyroid-specific genes, and weaken RAI response in PTC and ATC models [40,41]. PI3K-AKT-mTOR signaling contributes through survival, protein synthesis, and metabolic adaptation. PIK3CA, AKT, PTEN, and other PI3K-pathway alterations are enriched in PDTC and ATC [7]. In BRAF-mutant thyroid organoids, combined MAPK and PI3K inhibition restores follicular architecture and differentiation more effectively than either pathway alone [72,73]. PI3K-AKT signaling therefore acts mainly as a reinforcing and progression-associated program, rather than a universal initiating event.

### 3.2. Secondary Genetic Alterations Enable Progression Toward PDTC and ATC

Progression from DTC toward PDTC and ATC usually requires additional lesions beyond the initiating MAPK driver. TERT promoter mutations, TP53 inactivation, copy-number alterations, and mutations affecting PI3K-AKT signaling, SWI/SNF chromatin remodeling, and cell-cycle regulation become more common in high-grade tumors [7,13]. *BRAF^V600E^* and TERT promoter mutations cooperate clinically and identify tumors with higher recurrence and mortality risk [9]. These alterations do more than accelerate proliferation: they reduce lineage stability, increase genomic stress, and lower the probability that MAPK inhibition alone will restore durable thyroid function.

TERT promoter mutations illustrate how progression-associated genetics can intersect with dedifferentiation. TERT activation supports replicative immortality and is associated with aggressive clinicopathological features and poorer outcomes [8,13]. TP53 loss facilitates genomic instability and anaplastic transformation. Together, these events can shift the tumor from a differentiated, MAPK-driven state toward one in which thyroid lineage identity is less constrained and therapeutic plasticity is reduced [7,17]. Nevertheless, these alterations should be interpreted as factors that weaken lineage stability, not as direct functional proof of dedifferentiation; functional assessment still requires evaluation of thyroid-lineage markers and iodine handling.

### 3.3. Chromatin Remodeling Locks Tumor Cells into a Dedifferentiated State

Lineage loss becomes harder to reverse when chromatin architecture no longer supports thyroid transcriptional programs. SWI/SNF complex mutations can alter PAX8- and NKX2-1-associated regulatory regions, suppress thyroid differentiation genes, and reduce sensitivity to MAPK-directed redifferentiation [74,75]. ARID1A loss and other chromatin-remodeling defects can also promote thyroid tumor progression and treatment resistance [76]. These findings explain why some tumors fail to redifferentiate despite adequate inhibition of an upstream oncogenic pathway.

Epigenetic repression provides an additional layer of stabilization. DNA methylation and histone modifications can silence thyroid-lineage genes and NIS regulatory regions, while EZH2-dependent H3K27me3 may contribute to differentiation blockade in ATC models [77,78,79]. This chromatin barrier separates reversible pathway-dependent suppression from more stable lineage locking. Once the regulatory landscape has been remodeled, a redifferentiation regimen directed only at MAPK output may be insufficient and may need to be combined with interventions that restore chromatin accessibility or relieve epigenetic repression.

### 3.4. Metabolic and Cellular Stress Reinforce Lineage Loss

Metabolic and cellular stress programs further connect oncogenic signaling to lineage loss. Hypoxia, nutrient stress, oxidative stress, and endoplasmic reticulum stress can suppress iodine-handling genes and reshape tumor-cell behavior. HIF-1α/YAP signaling can rewire glucose and iodine metabolism and promote PTC progression [39]. NOX4-mediated oxidative stress can inhibit NIS and impair radioiodine response [77,78], while hypoxia-associated non-coding RNA regulation and DNMT1-mediated methylation may further influence NIS expression [80]. In this section, these processes are considered tumor-cell-intrinsic stress responses that weaken lineage programs; the role of CAFs, TAMs, cytokines, and extracellular matrix remodeling in maintaining these stresses is addressed in Section 5.

These stress programs are not merely passive consequences of tumor growth. Persistent low oxygen, altered nutrient availability, and oxidative pressure select for cells that can tolerate metabolic and inflammatory stress. Such cells may remain partly dependent on the original driver pathway, but their phenotype is increasingly shaped by stress adaptation and loss of lineage constraints. This provides one explanation for why partial pathway inhibition may not fully reverse dedifferentiation, even when the initiating oncogenic signal is reduced.

### 3.5. Dedifferentiation Is Initially Reversible but May Become Stabilized

The strongest evidence for reversibility comes from redifferentiation studies. MEK, RAF, and combined RAF-MEK inhibition can restore iodine uptake in subsets of RAI-refractory thyroid cancers [44,45,81,82,83,84,85,86,87]. These responses show that, in some tumors, lineage suppression remains pharmacologically reversible. However, only a fraction of patients regain uptake sufficient for therapeutic ^131^I administration, and the durability of restored differentiation varies across tumors and treatment contexts.

**Figure 2:**
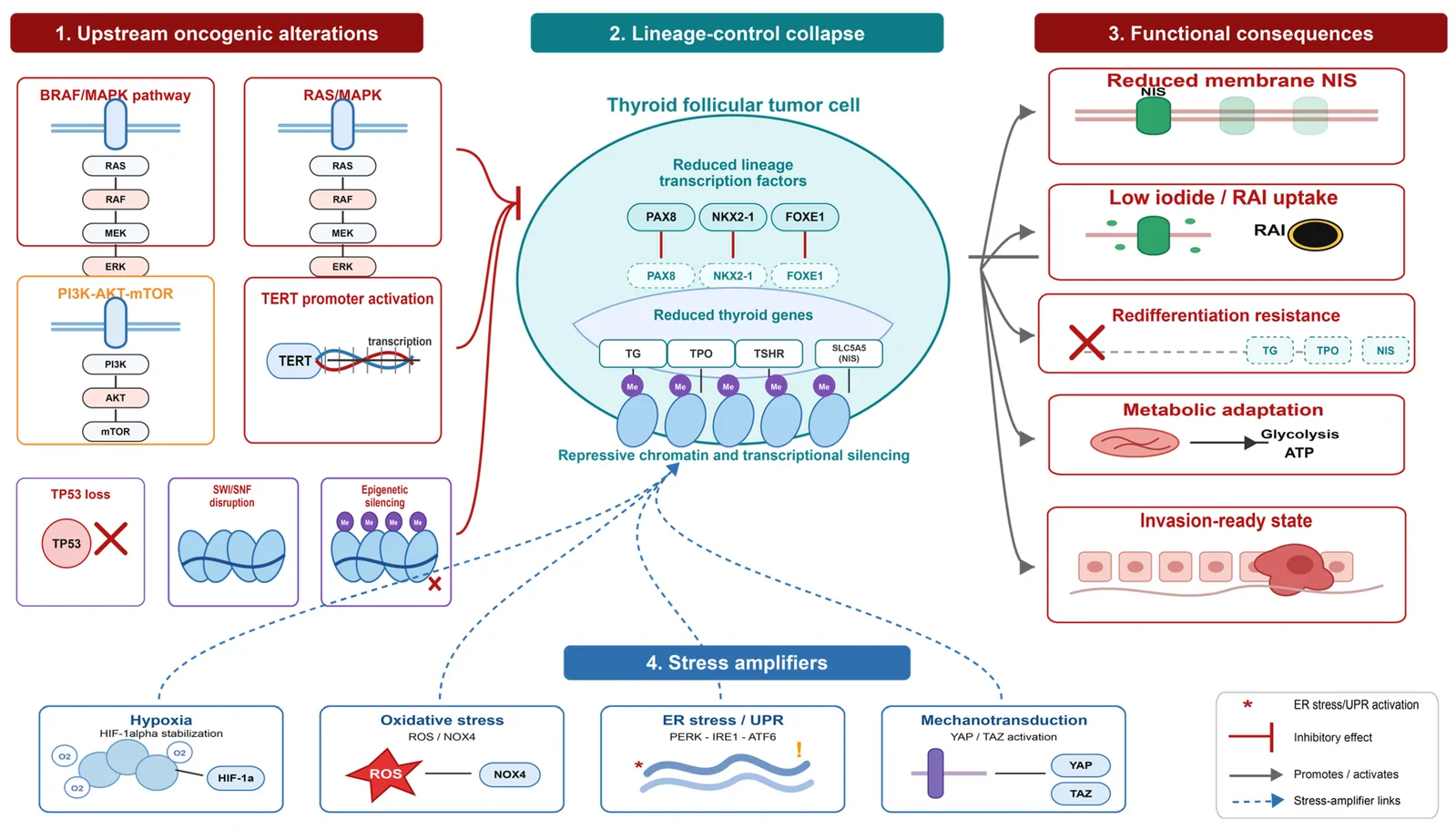
Tumor-intrinsic mechanisms of thyroid cancer dedifferentiation. Oncogenic MAPK and PI3K-AKT signaling, TERT promoter activation, TP53 loss, SWI/SNF disruption, and epigenetic silencing converge on reduced thyroid-lineage transcription factors and thyroid-function genes. Tumor-cell stress and mechanical-response programs, including hypoxia, oxidative stress, ER stress, and mechanotransduction, reinforce lineage-control collapse and promote impaired NIS membrane localization, low radioiodine uptake, redifferentiation resistance, metabolic adaptation, and invasion-ready phenotypes. Abbreviations: NIS, sodium–iodide symporter; RAI, radioactive iodine. This figure was created using Adobe Illustrator 2025 (Version 29). Certain biological motifs were manually redrawn and modified by the authors with reference to materials by Servier from Servier Medical Art, accessed through Bioicons. The original source materials are licensed under the Creative Commons Attribution 3.0 Unported license (CC BY 3.0; https://creativecommons.org/licenses/by/3.0/, accessed on 25 June 2026), and all modifications and final figure composition were made by the authors. In the ER stress/UPR module, the red asterisk denotes ER stress/UPR activation, whereas the yellow exclamation mark is a visual emphasis marker without quantitative meaning.

A useful model is that early dedifferentiation is dominated by reversible signaling output, whereas later disease accumulates genetic, chromatin, and stress-adaptation changes that make lineage loss more stable. Microenvironmental feedback may further maintain this low-differentiation state, but its detailed mechanisms are discussed in the following sections. This distinction supports a mechanism-guided treatment strategy: tumor-intrinsic pathway inhibition may open a redifferentiation window, whereas durable restoration of iodine handling may require simultaneous control of epigenetic barriers and microenvironmental feedback. The tumor-intrinsic mechanisms discussed above are summarized in Figure 2.

## 4. How Dedifferentiating Tumor Cells Remodel the Microenvironment

Dedifferentiation reshapes tumor-cell morphology, transcription, and iodine handling, and it also changes how malignant cells communicate with immune cells, fibroblasts, extracellular matrix, and distant tissues. As differentiation declines, tumor cells acquire inflammatory secretory programs, immune-evasion capacity, matrix-remodeling activity, and the ability to condition metastatic niches [17,18,19,20,21,22,23,24,25,26,27,68]. This section focuses on how dedifferentiating tumor cells transmit signals outward to remodel the surrounding ecosystem. The reciprocal feedback that stabilizes lineage loss is discussed in Section 5.

### 4.1. Dedifferentiation Establishes an Inflammatory Secretory State

Hypoxia, nutrient deprivation, endoplasmic reticulum stress, and sustained MAPK signaling can alter the secretome of thyroid cancer cells. Under metabolic or ER stress, thyroid cancer cells increase IL-6 and CXCL8, and exogenous IL-6 or CXCL8 can reduce thyroid-specific gene expression and RAI uptake [26]. Sustained *BRAF^V600E^* signaling can also promote TGF-β secretion. TGF-β suppresses NIS and increases invasion while influencing fibroblast activation, matrix deposition, and immune-cell function [38]. These tumor-derived signals form an initial communication layer that can recruit or reprogram stromal and immune populations. Here, the emphasis is on signal production by tumor cells; the feedback mechanisms that maintain dedifferentiation are addressed in Section 5.

### 4.2. Recruitment and Functional Reprogramming of Tumor-Associated Macrophages

TAMs are a prominent component of aggressive thyroid cancer. Increased macrophage density correlates with extrathyroidal extension, nodal metastasis, PDTC/ATC histology, and poor outcome [21,22,56]. Mechanistic work shows that macrophage-derived CXCL16 can activate CXCR6-AKT signaling in PTC cells, increasing migration and invasion, while tumor-derived signals reciprocally promote immunoregulatory macrophage features [29]. PDCD4 loss in tumor cells can enhance eIF4A-dependent expression of immunosuppressive mediators and enrich TYMP-positive macrophages in poorly differentiated regions [27].

These studies support a bidirectional model rather than a one-way recruitment model. Dedifferentiating tumor cells create a chemokine, cytokine, and vesicular environment that attracts or polarizes macrophages, whereas macrophages feed back to promote invasion and immune suppression. Tumor-derived exosomal lncRNA FGD5-AS1 has been reported to promote M2-like macrophage polarization and to enhance thyroid cancer cell migration and invasion in co-culture models [59]. In a complementary endothelial model, FGD5-AS1-containing thyroid cancer exosomes enhanced angiogenesis and vascular permeability through the miR-6838-5p/VAV2 axis [88]. Nevertheless, direct evidence that TAMs alone suppress NIS expression or restore iodine uptake when reprogrammed remains limited. TAMs are therefore best considered an important amplifier of the dedifferentiation-associated niche rather than a proven universal cause of thyroid functional loss.

### 4.3. Fibroblast Activation and Extracellular Matrix Remodeling

CAF expansion is a consistent feature of advanced and dedifferentiated thyroid cancer. Spatial and single-cell studies identify fibroblast states associated with extracellular-matrix organization, TGF-β signaling, inflammatory signaling, and metabolic rewiring [18,19,20,24,25,27,28,63,68]. Functionally, CAFs can promote tumor proliferation, invasion, EMT-like features, and immune exclusion. Fibroblast activation protein (FAP) and fibronectin 1 (FN1) expression, together with TGF-β-associated stromal programs, is linked to aggressive thyroid cancer behavior and immunosuppression [63].

Fibroblast remodeling affects dedifferentiation through several routes. CAF-derived lactate can suppress thyroid differentiation and iodine uptake through TGF-β/Smad signaling [28]. Matrix stiffness and mechanotransduction may activate YAP/TAZ and other pathways that reinforce malignant plasticity [66,67]. Thyroid tumor cell-fibroblast interactions can also generate extracellular vesicles (EVs) with CD147 and matrix-remodeling features, which promote MMP2/MMP9 activity and a migratory phenotype in experimental models [57,58]. These findings indicate that EVs may convert local tumor-stroma interactions into transferable signals that promote ECM degradation. The consequences of lactate, matrix stiffness, and mechanical signaling for the persistence of lineage loss are discussed in Section 5.

### 4.4. Additional Immune Compartments and Immune Escape

Other immune compartments may also influence dedifferentiation-associated phenotypes. Mast cells can promote thyroid cancer EMT, stemness, and tumorigenicity through CXCL8-AKT-Slug signaling [42]. This mechanism has strong support for invasion and stemness, but it does not establish direct loss of iodine-handling function. T-cell dysfunction is frequently observed in dedifferentiated and anaplastic tumors, where immune-checkpoint expression and myeloid-dominated suppression increase [17,18,19,20,21,22,23,24,25].

EV-mediated communication may contribute to this immune dysfunction. In metastatic PTC, tumor-derived exosomal miR-519e-5p can be transferred to CD8+ T cells and reduce cytotoxic function through a NOTCH2-related mechanism, with increased PD-1 and reduced granzyme B expression [60]. These findings connect tumor-derived EVs with immune escape and distant metastasis. However, mast-cell and T-cell programs are currently better supported as regulators of invasive plasticity and immune suppression than as direct controllers of NIS expression or RAI uptake.

### 4.5. Extracellular Vesicles as Emerging Mediators of Tumor–Microenvironment Communication

Extracellular vesicles are membrane-bound particles that carry proteins, lipids, metabolites, and nucleic acids between cells. Because the biogenesis of an isolated vesicle preparation is often difficult to establish, the generic term extracellular vesicle (EV) is preferred unless an endosomal origin has been demonstrated [62]. This distinction is important because many thyroid cancer studies use the term exosome for heterogeneous vesicle preparations.

In thyroid cancer, EVs can act on macrophages, fibroblasts, T cells, endothelial cells, and distant stromal cells. Their cargo may alter recipient-cell transcription, cytokine secretion, matrix remodeling, and immune function. The available evidence supports several interconnected roles: EVs can promote M2-like macrophage polarization [59], stimulate fibroblast-associated MMP activity [57,58], and impair CD8+ T-cell cytotoxicity [60]. EVs may therefore provide a route through which dedifferentiating tumor cells propagate inflammatory, immunosuppressive, and matrix-remodeling signals beyond the cells that initially generated them.

The relationship between EVs and thyroid cancer dedifferentiation remains incompletely defined. Most studies have focused on proliferation, EMT, invasion, immune escape, or metastasis rather than on direct functional readouts of thyroid identity. Few studies have simultaneously measured SLC5A5/NIS, TSHR, PAX8, TG, TPO, NIS membrane localization, and radioiodine uptake after EV exposure. Thus, endogenous tumor-derived EVs should currently be viewed as potential mediators of a dedifferentiation-associated ecosystem, not as an established direct cause of NIS loss or RAI resistance. In contrast, engineered EVs have been explored as delivery vehicles for tyrosine kinase inhibitors that can restore NIS expression and radioiodine uptake in RAI-refractory ATC models; this therapeutic application should be distinguished from the pathogenic role of endogenous EVs [61].

### 4.6. Extension from the Local Microenvironment to the Premetastatic Niche

Dedifferentiating tumor cells may also condition lymphatic and distant niches. Exosomal miR-17-5p has been reported to act on lung fibroblasts, activate NF-κB-related inflammatory signaling, and increase IL-6 and IL-8 secretion, thereby promoting a pulmonary microenvironment favorable for thyroid cancer colonization [89]. Chemokine axes such as CXCL16/CXCR6, CCL21/CCR7, and CXCL12/CXCR4 or CXCR7 provide additional routes through which local inflammation and immune remodeling may become linked to dissemination [29,52,90,91,92].

The evidence remains uneven. Some EV and chemokine mechanisms have in vivo support, whereas others remain associative or model-dependent. These circuits are most relevant to this review when they connect lineage instability with immune suppression, lymphatic access, distant colonization, or potential treatment resistance. Lymph-node metastasis itself should not be treated as a surrogate for functional dedifferentiation unless thyroid-lineage markers, NIS localization, iodine uptake, or RAI response are also assessed.

## 5. Microenvironmental Feedback That Stabilizes Dedifferentiation

Tumor-intrinsic signaling through MAPK, PI3K-AKT, and TGF-β can initiate dedifferentiation, but the persistence of this state may not depend solely on the original driver. RAF or MEK inhibition can restore iodine uptake in some RAI-refractory tumors, indicating that early dedifferentiation is partly reversible [44,45,69,70,81,82,83,84]. Yet only some patients regain uptake sufficient for treatment, and restored differentiation may not be durable. CAFs, TAMs, inflammatory cells, hypoxia, and abnormal extracellular matrix may provide external feedback that converts transient lineage suppression into a more stable low-differentiation state.

### 5.1. CAF Metabolic Reprogramming Sustains Lineage Loss Through Lactate

CAF metabolic reprogramming currently offers one of the strongest functional links between the thyroid cancer microenvironment and dedifferentiation. Spatial transcriptomics identified glycolytic CAFs in poorly differentiated regions, with increased ZFP57 and pyruvate kinase M2 (PKM2) expression [20,28,68]. Functional experiments showed that ZFP57 promotes PKM2 expression and lactate production in CAFs. CAF-conditioned medium increased PTC-cell proliferation and invasion while reducing thyroid differentiation genes, RAI uptake, and iodine retention. CAF-derived lactate activated tumor-cell TGF-β/Smad2/3 signaling, forming a ZFP57-PKM2-lactate-TGF-β/Smad2/3 feedback axis [28].

The value of this work is that it connects spatially identified CAF states to metabolic assays, conditioned medium, gene regulation, iodine uptake, and animal experiments. Lactate moves from being a regional correlate of poor differentiation to a candidate functional mediator of lineage control. In other tumors, lactate can also shape angiogenic and immunoregulatory TAM programs through HIF-1α [64]. In thyroid cancer, CAF-derived lactate may therefore act on both malignant cells and myeloid cells, linking metabolic feedback to immune suppression. Direct evidence remains limited to selected spatial samples and experimental models, and the proposed resveratrol sensitivity of the ZFP57-PKM2 axis will need more specific genetic and pharmacologic validation.

### 5.2. IL-6, CXCL8, and TGF-β Form an Inflammatory Dedifferentiation Circuit

Inflammatory cytokines provide a second route through which microenvironmental stress can stabilize lineage loss. IL-6 and CXCL8 induced by hypoxia and endoplasmic reticulum stress reduce thyroid-specific genes and RAI uptake [26]. TGF-β signaling suppresses NIS and promotes invasion downstream of *BRAF^V600E^*, MED16 loss, or Smad7 destabilization [38,40,41]. These cytokines can be produced by tumor cells, fibroblasts, macrophages, or endothelial cells, making source attribution essential.

Rather than a single cytokine cascade, the data point to an inflammatory circuit in which tumor stress, stromal activation, and immune infiltration reinforce one another. Such a circuit could explain why redifferentiation after MAPK inhibition is incomplete in some tumors. If inflammatory or TGF-β-driven suppression persists after oncogenic output falls, thyroid genes may recover only partially and NIS may not reach a functional membrane state.

### 5.3. TAMs May Amplify the Dedifferentiated Niche, but Direct Evidence Remains Incomplete

TAMs are repeatedly associated with poorly differentiated and anaplastic thyroid cancer, and several mechanisms link them to invasion and immune suppression [21,22,27,29,32,56]. CXCL16/CXCR6-AKT signaling increases PTC-cell migration, and TIMP1-PI3K/AKT signaling can promote thyroid cancer progression through macrophage phenotype polarization [29,32]. PDCD4 loss links tumor-cell translational control to TYMP-positive macrophage enrichment [27].

These findings support the idea that TAMs amplify a dedifferentiated niche. However, the field still lacks decisive experiments showing that macrophage depletion or reprogramming restores NIS membrane localization, iodine uptake, or RAI response in vivo. Until such data are available, TAMs should be presented as strong candidates for stabilizing invasion and immune escape, and as plausible contributors to dedifferentiation rather than proven direct drivers of thyroid functional loss.

### 5.4. Mast-Cell Feedback Primarily Promotes EMT and Stemness

Mast-cell signaling illustrates the importance of readout specificity. Mast-cell-derived CXCL8 can activate AKT-Slug signaling, increase EMT and stemness, and enhance tumorigenicity in thyroid cancer models [42]. These effects are relevant to plasticity and progression, but they do not establish direct suppression of thyroid differentiation genes or iodide uptake. Mast-cell feedback should therefore be discussed as a mechanism of malignant plasticity that may intersect with dedifferentiation, not as a validated cause of thyroid-lineage loss.

### 5.5. ECM Stiffness, Hypoxia, and Mechanical Signaling Stabilize Malignant Plasticity

At the microenvironmental level, matrix stiffness, hypoxia, and mechanical signaling may prolong these tumor-cell stress programs after the initiating oncogenic signal has been reduced. YAP/TAZ-dependent mechanotransduction can cooperate with growth-factor and hypoxia pathways to support invasion, metabolic rewiring, and therapy resistance [66,67]. In this context, PKM2-related metabolic programs and hypoxia-associated epigenetic changes may contribute to persistent NIS suppression [43,80]. Thus, Section 3.4 describes the intracellular stress response, whereas this subsection addresses the external conditions that sustain it.

These stress and mechanical signals may persist even after MAPK output is pharmacologically reduced. They provide a plausible explanation for unstable or incomplete redifferentiation, particularly in fibrotic, hypoxic, or myeloid-rich lesions. Stronger in vivo models are needed to determine whether targeting matrix stiffness, hypoxia, or metabolic stress can improve lesion-level radioiodine dosimetry.

### 5.6. From Reversible Dedifferentiation to Ecosystem Locking

Together, these observations suggest a transition from reversible pathway-driven suppression to ecosystem locking. In early or partially dedifferentiated tumors, RAF or MEK inhibition may restore thyroid-lineage transcription and iodine uptake. With progression, chromatin remodeling, CAF metabolism, TAM-associated inflammation, hypoxia, and matrix remodeling may sustain the phenotype even when the initiating signal is inhibited. This does not mean dedifferentiation is irreversible. It means that durable reversal may require simultaneous disruption of malignant-cell programs and the microenvironmental feedback that maintains them. These reciprocal tumor–microenvironment interactions are summarized in Figure 1 and Table 2.

## 6. From Local Plasticity to Lymphatic Metastasis

Cervical lymph-node metastasis is one of the most common routes of spread in PTC, but nodal metastasis is not synonymous with dedifferentiation. Many PTCs that retain thyroid-lineage markers and RAI avidity can metastasize early to regional lymph nodes, whereas some PDTCs and ATCs mainly show local invasion or hematogenous spread. Progression from local tumor growth to lymph-node metastasis requires at least three related but distinct processes: acquisition of invasive capacity, expansion of lymphatic access, and responsiveness to lymphatic or nodal chemotactic cues.

For this review, nodal metastasis should be framed as a consequence of invasion, lymphangiogenesis, chemokine responsiveness, and microenvironmental remodeling. It may accompany dedifferentiation in aggressive disease, but it cannot be used as a surrogate for loss of thyroid-lineage function.

### 6.1. Lymph-Node Metastasis Is Related to but Not Equivalent to Dedifferentiation

Dedifferentiation primarily denotes loss of thyroid-lineage and iodine-handling programs, whereas lymph-node metastasis requires invasion, lymphatic entry, survival in transit, and nodal colonization. EMT, TGF-β signaling, hypoxia, inflammatory pathways, and MAPK/PI3K-AKT activity can contribute to both phenotypes, but the phenotypes are not interchangeable [37,38,39,40,41,42]. *BRAF^V600E^*-TGF-β signaling can suppress NIS while promoting EMT and invasion [38], whereas mast-cell-derived CXCL8 promotes EMT and stem-like features without directly demonstrating NIS loss or impaired iodine uptake [42]. Nodal disease should therefore be treated as a possible output of an aggressive ecosystem, not as a definition of functional dedifferentiation.

### 6.2. Invasion, Lymphangiogenesis, and Chemokine Cues

Local invasion opens access to lymphatic vessels through matrix metalloproteinases, integrin and focal-adhesion remodeling, cytoskeletal changes, and CAF/TAM-mediated tissue remodeling [32,41,63,96]. VEGF-C/VEGF-D signaling through VEGFR-3 is associated with lymphatic vessel density and nodal metastasis in PTC [52,53,54]. Chemokine cues may then guide dissemination: CCR7/CCL21 signaling has been linked to thyroid-carcinoma migration and nodal homing [90], while CXCL12 receptors regulate migration through biologically distinct CXCR4 and CXCR7 programs [91,92]. These pathways explain metastatic access and positioning, but they do not by themselves establish loss of thyroid function or RAI resistance.

### 6.3. Clinical Interpretation of Nodal Disease in the Context of RAI Resistance

The prognostic meaning of nodal metastasis depends on metastatic-node size and number, extranodal extension, age, and overall disease burden [51,55,93,98]. Microscopic nodal disease may have limited effects on survival, whereas bulky or extranodal disease carries a higher recurrence risk. In a review centered on dedifferentiation and RAI resistance, nodal metastasis is best interpreted as a clinically important but non-specific consequence of invasion, lymphangiogenesis, chemokine responsiveness, and microenvironmental remodeling. Future experiments should measure nodal dissemination together with lineage markers, NIS localization, and functional iodine uptake rather than using nodal status as a surrogate endpoint.

## 7. Radioiodine Resistance as an Ecosystem-Level Phenotype

### 7.1. Radioiodine Resistance Reflects Multilevel Failure of the Iodine-Handling Machinery

RAI therapy requires tumor cells to uptake, retain, and organify iodide. This process depends not only on NIS/SLC5A5 transcription, but also on NIS glycosylation, basolateral membrane trafficking, stable membrane residence, and downstream organification by TPO, TG, and hydrogen peroxide-generating systems. RAI resistance therefore cannot be reduced to low NIS mRNA. Some thyroid cancers retain NIS protein that is largely intracellular and transport-incompetent [4,6]. PBF/PTTG1IP (pituitary tumor-transforming gene 1-interacting protein) phosphorylation promotes removal of NIS from the plasma membrane, while SRC inhibition or modulation of PBF phosphorylation can increase membrane NIS and iodide uptake [99]. NIS membrane residence is also regulated by an SRC-RAC1-PAK1-PIP5K-EZRIN cytoskeletal axis [100]. Adaptor protein complex 2 (AP2)-mediated NIS endocytosis is pharmacologically tractable, and chloroquine combined with histone deacetylase inhibition can increase plasma-membrane NIS and radionuclide uptake in vivo [95]. RAI resistance therefore includes transcriptional, post-translational, trafficking, endocytic, and retention defects.

### 7.2. Tumor-Intrinsic Programs Establish the Initial RAI-Refractory State

Sustained MAPK signaling is a central intrinsic driver of thyroid-lineage suppression and RAI resistance. *BRAF^V600E^* reduces PAX8, NKX2-1, NIS, TG, TPO, and TSHR expression, and adequate ERK inhibition can partially restore differentiation genes and iodide uptake [69,70]. PI3K-AKT, TGF-β/Smad signaling, and HER3 feedback activation weaken the redifferentiation response to MAPK inhibition [38,40,41,71,72]. SWI/SNF defects and enhancer closure can push differentiation genes into a more refractory chromatin state [74,75,76]. NOX4-mediated oxidative stress and DNA damage may further suppress thyroid differentiation through epigenetic mechanisms [77,78]. At the post-transcriptional level, the miR-17-92 cluster can reduce NIS, TPO, TG, PAX8, and NKX2-1, while CRISPR-based silencing partially restores differentiation in ATC cells [101]. Tumor-intrinsic alterations establish the molecular basis of RAI resistance, but reversibility depends on genetic, epigenetic, and feedback contexts.

### 7.3. Hypoxia, Inflammation, and CAF Metabolism Reinforce Iodine-Handling Failure

Microenvironmental stress reinforces iodine-handling failure. Hypoxia and ER stress increase IL-6 and CXCL8, which suppress thyroid genes and reduce RAI uptake [26]. HIF-1α/YAP and PKM2-associated metabolic programs rewire glucose and iodine metabolism [39,43]. CAF-derived lactate activates TGF-β/Smad2/3 signaling and reduces iodine uptake [28]. Hypoxia inhibition can restore iodine uptake in experimental models through a hsa_circ_0023990-miR-448-DNMT1-NIS axis [80]. These mechanisms suggest that RAI resistance is sustained by both malignant-cell lineage loss and external microenvironmental pressure.

### 7.4. Why MAPK Inhibition Alone May Not Sustain Redifferentiation

MAPK inhibition can reopen a redifferentiation window, but it may not be sufficient to maintain it. Feedback HER3/NRG1 signaling can reactivate ERK [71]. PI3K-AKT and TGF-β pathways can continue to suppress differentiation and promote survival [38,40,41,72]. Chromatin remodeling defects may make thyroid genes less accessible [74,75,76]. CAF-derived lactate, inflammatory cytokines, hypoxia, and abnormal matrix can persist after pathway inhibition and continue to impair NIS expression or membrane localization [26,28,39,43,63,80]. RAI resistance is therefore best considered an ecosystem-level phenotype. Durable redifferentiation may require combined targeting of MAPK output, NIS trafficking, epigenetic locking, and microenvironmental feedback.

## 8. Therapeutic Targeting of Dedifferentiation and Its Microenvironment

### 8.1. Genotype-Guided MAPK Inhibition Is the Most Clinically Advanced Redifferentiation Strategy

The goal of redifferentiation therapy is not simply tumor shrinkage. It is to restore thyroid-lineage programs and effective iodine uptake long enough for RAI-refractory lesions to become treatable with ^131^I. The most clinically advanced strategy is short-course MAPK inhibition selected by genotype. Selumetinib, dabrafenib, and vemurafenib can restore iodine uptake in subsets of RAI-refractory thyroid cancer, but responses vary by genotype [44,45,81]. RAS-mutant tumors often show greater sensitivity to MEK inhibition, whereas *BRAF^V600E^* tumors may require stronger RAF-MEK suppression to overcome high MAPK output and feedback reactivation [44,70,71,83,86].

In metastatic RAI-refractory *BRAF^V600E^*-mutant DTC, dabrafenib plus trametinib restored abnormal iodine uptake in most evaluable patients, and the partial response rate six months after ^131^I was 38% [85]. In RAS-mutant disease, trametinib followed by ^131^I also showed the possibility of restored uptake and disease control [86]. These studies support a treatment sequence of short-course targeted inhibition, uptake assessment, dosimetry-based selection, and ^131^I therapy, rather than indefinite use of MAPK inhibitors as conventional cytotoxic therapy [83,85,86,87].

Redifferentiation therapy should not be generalized to all DTC patients. In the phase III ASTRA trial, selumetinib added to adjuvant RAI did not significantly improve complete remission in high-risk DTC before the establishment of RAI-refractory disease [102]. MAPK inhibition is therefore most appropriate for patients with proven inadequate iodine uptake and potentially reversible differentiation suppression, not as routine RAI sensitization for all high-risk tumors.

### 8.2. Epigenetic and NIS-Trafficking Interventions May Overcome Incomplete Redifferentiation

Some tumors fail to restore iodine uptake despite adequate MAPK inhibition, probably because of chromatin locking or defective NIS trafficking. SWI/SNF defects can close PAX8- and NKX2-1-associated regulatory regions and reduce sensitivity to MAPK redifferentiation [74]. HDAC inhibition combined with MAPK inhibition can increase differentiation-gene expression in *BRAF^V600E^* thyroid cancer cells, although evidence is mostly preclinical [103]. EZH2 inhibition can reduce H3K27me3-associated lineage silencing, restore SLC5A5, NKX2-1, TSHR, FOXE1, and TPO expression, and partially increase iodide uptake in ATC cells, with stronger effects when combined with MEK inhibition [79].

Another strategy is to deliver expressed NIS to the membrane and stabilize it there. Modulating PBF/PTTG1IP phosphorylation, the SRC-RAC1-PAK1-PIP5K-EZRIN axis, or AP2-mediated NIS endocytosis can improve membrane NIS and iodide uptake [95,99,100]. PI3K-AKT inhibition may also influence NIS membrane trafficking [73]. These interventions are not redundant with MAPK inhibition. One restores lineage transcription; the other improves NIS localization and function. Future combinations should assess NIS expression, membrane localization, and functional uptake before selecting the intervention.

### 8.3. Targeting CAF Metabolism, Hypoxia, and TGF-β May Prevent Ecosystem-Mediated Relapse

If CAF, hypoxic, and inflammatory signals persist, tumor cells may re-enter a poorly differentiated state after MAPK inhibition is stopped. The main value of microenvironmental therapy may be to extend the redifferentiated window and prevent renewed loss of RAI sensitivity, rather than to induce redifferentiation alone.

CAFs in poorly differentiated regions can increase glycolysis and lactate release through ZFP57-PKM2, activating tumor-cell TGF-β/Smad2/3 signaling and reducing iodine uptake; resveratrol suppressed this metabolic feedback in the experimental system [28]. A FAP-FN1-TGF-β axis is also implicated in stromal remodeling and immunosuppression in aggressive thyroid cancer [63]. Hypoxia inhibition may improve iodine uptake through the hsa_circ_0023990-miR-448-DNMT1-NIS axis [80]. These findings nominate CAF metabolism, lactate transport, FAP, TGF-β, and hypoxia pathways as combination targets. Their current limitation is translational: clinical evidence that these interventions restore therapeutic radioiodine dosimetry is still lacking.

### 8.4. TAM Reprogramming and Immune-Checkpoint Blockade Address Tumor Control Rather than Proven Redifferentiation

TAMs are attractive therapeutic candidates in poorly differentiated thyroid cancer. CXCL16/CXCR6-AKT and TIMP1-PI3K/AKT signaling can promote invasion and immunoregulatory macrophage phenotypes [29,32]. PDCD4 loss is associated with enrichment of TYMP-positive TAMs in poorly differentiated regions [27]. Blocking macrophage recruitment, interfering with CXCL16 or TIMP1 signaling, or reprogramming TAMs toward antitumor states could weaken the myeloid niche that supports dedifferentiation. The key limitation is that TAM depletion or reprogramming has not yet been shown to restore NIS membrane localization or in vivo RAI uptake.

Immune-checkpoint inhibition has shown clinical activity in ATC. Spartalizumab produced an objective response rate of 19%, with responses concentrated in PD-L1-positive tumors [104]. Retrospective data suggest that adding pembrolizumab after kinase-inhibitor progression can produce responses, although sample size and selection bias limit interpretation [105]. Dabrafenib plus trametinib has meaningful activity in *BRAF^V600E^*-mutant ATC [106], and immunocompetent mouse models suggest that BRAF inhibition may cooperate with PD-L1 blockade [107]. Tumor shrinkage or increased T-cell infiltration, however, is not equivalent to thyroid redifferentiation. Immunotherapy should be framed as a strategy to control poorly differentiated tumors and disrupt immunosuppressive niches, not as a proven RAI-sensitizing therapy.

### 8.5. Combination Therapy Should Be Guided by Functional Defects and Pharmacodynamic Endpoints

Future therapeutic design should not simply combine drugs. It should match combinations to the level at which RAI resistance is maintained. Tumors with high MAPK output but accessible lineage chromatin may benefit from short-course RAF or MEK inhibition. Tumors with EZH2- or SWI/SNF-associated epigenetic locking may require chromatin-modifying therapy. Tumors with NIS expression but poor membrane localization may need trafficking or endocytosis interventions. Tumors enriched for CAFs, hypoxia, and TAMs may require microenvironmental combinations.

**Figure 3:**
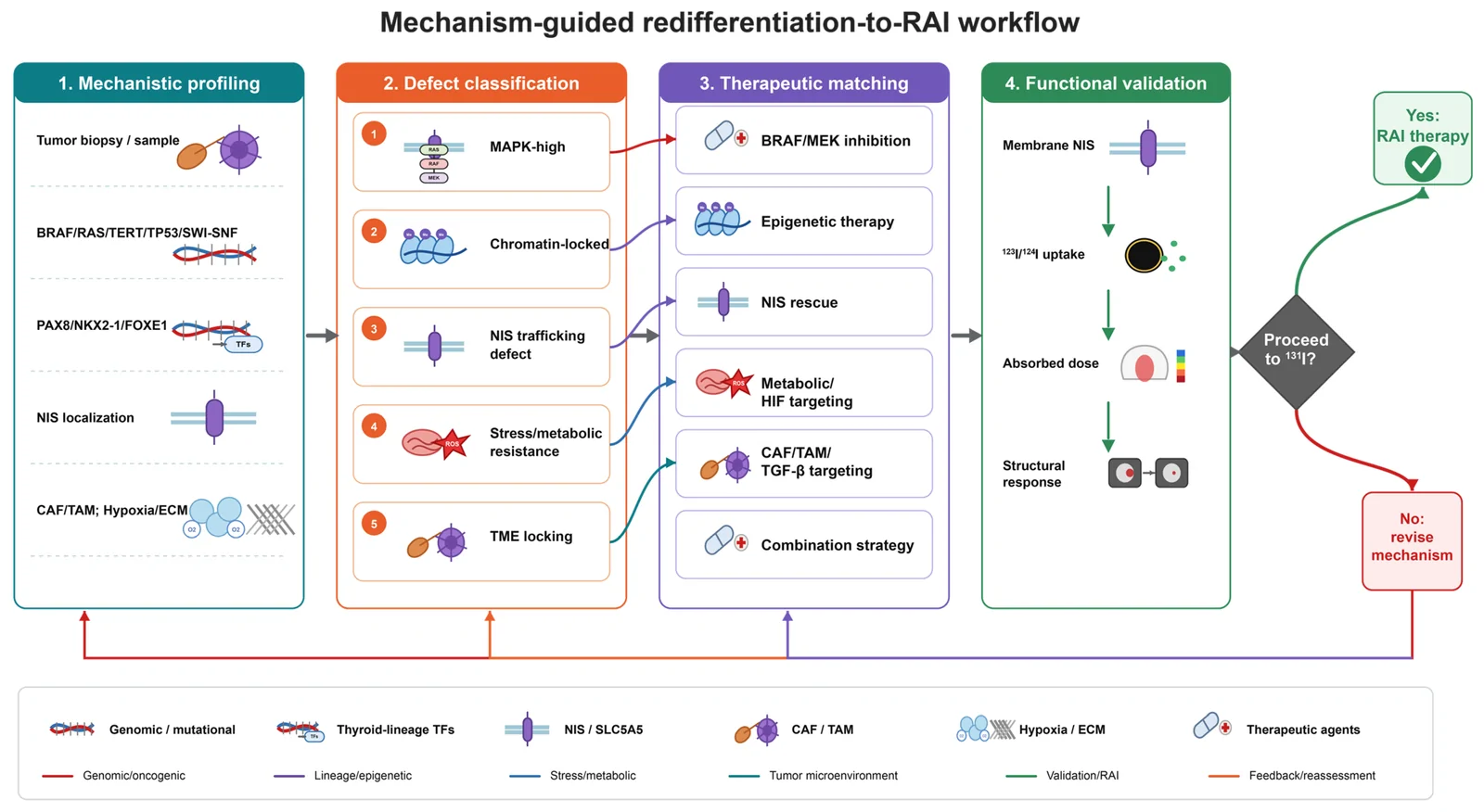
Mechanism-guided redifferentiation-to-radioiodine workflow. Mechanistic profiling of genotype, thyroid-lineage programs, NIS localization, and microenvironmental features can classify dominant resistance states, including MAPK-high signaling, chromatin locking, NIS trafficking defects, stress or metabolic resistance, and tumor–microenvironment locking. Therapeutic matching should then be followed by functional validation of membrane NIS, ^123^I/^124^I uptake, lesion absorbed dose, and structural response before proceeding to ^131^I therapy; failure to restore iodine handling should prompt mechanism reassessment rather than empirical RAI treatment. Abbreviations: NIS, sodium–iodide symporter; RAI, radioactive iodine. This figure was created using Adobe Illustrator 2025 (Version 29). Certain biological motifs were manually redrawn and modified by the authors with reference to materials by Servier from Servier Medical Art, accessed through Bioicons. The original source materials are licensed under the Creative Commons Attribution 3.0 Unported license (CC BY 3.0; https://creativecommons.org/licenses/by/3.0/, accessed on 25 June 2026), and all modifications and final figure composition were made by the authors.

Clinical endpoints should also move beyond tumor shrinkage. Evaluation should include thyroid differentiation scores, NIS protein and membrane localization, pre- and post-treatment ^123^I or ^124^I uptake, lesion-absorbed dose, structural response after ^131^I, and duration of response [83,85,86,87]. Microenvironmental targeting will become a true redifferentiation strategy only if it increases tumor iodine dose and improves RAI outcome in vivo. A mechanism-guided therapeutic framework is summarized in Figure 3 and Table 3; the clinically evaluated redifferentiation studies are compared separately in Table 4.

## 9. Evidence Gaps and Future Directions

The available literature supports a model in which thyroid cancer dedifferentiation is shaped by malignant cells, CAFs, TAMs, hypoxia, and inflammatory signaling. Several gaps remain. Many studies are still correlative. Enrichment of CAFs, TAMs, immunosuppressive cells, TGF-β, IL-6/CXCL8, lactate metabolism, or hypoxia in poorly differentiated regions indicates association, but it does not establish whether these factors initiate dedifferentiation, maintain it, or arise as consequences of progression [18,19,20,21,22,23,26,27,28,29,32,54,63]. Time-resolved models, conditional genetic perturbation, and reconstructed cell-interaction systems are needed to separate mechanisms that induce lineage loss from those that adapt to an already dedifferentiated niche.

Model systems are another limitation. ATC, PDTC, and RAI-refractory DTC are often used to study dedifferentiation, but terminal disease states do not fully represent early PTC-to-PDTC evolution. Cell lines and immunodeficient mouse models are useful for mechanism testing but often lack intact immune context, stromal architecture, and follicular differentiation. More informative systems would include immunocompetent *BRAF^V600E^* or TERT-promoter-mutant models, organoid-stroma-immune co-cultures, and dynamic models that simulate RAI pressure and withdrawal after redifferentiation therapy [70,74,83,107].

The field also needs harmonized endpoints. Studies variously use thyroid differentiation scores, SLC5A5/NIS expression, PAX8/NKX2-1/FOXE1 programs, EMT markers, proliferation, or RAI uptake [3,4,14,83]. Future work should evaluate transcriptional differentiation, NIS protein and membrane localization, ^123^I/^124^I uptake, ^131^I absorbed dose, and structural response in the same framework. For microenvironmental therapies, reducing invasion, immune suppression, or cell infiltration is not enough. The decisive test is whether these changes restore an effective RAI treatment window [79,80,85,86,87,95,99,100,101].

Finally, prognostic mechanisms must be separated from treatable mechanisms. CAF metabolism, TAM enrichment, TGF-β signaling, and hypoxia may explain the relationship among invasion, nodal metastasis, and RAI resistance, but not every prognostic pathway is a suitable therapeutic target [28,29,32,43,63]. The most translatable mechanisms are those that persist in poorly differentiated or RAI-resistant lesions, can be perturbed experimentally to reverse differentiation loss, and increase functional iodine uptake or therapeutic response in vivo. This standard would move the field from describing a complex microenvironment toward identifying reversible ecosystem dependencies.

## 10. Conclusions

Thyroid cancer dedifferentiation is not fully explained by a single driver mutation or one tumor-cell pathway. It reflects loss of tumor-cell lineage programs maintained by external microenvironmental feedback. BRAF/MAPK, TERT, TP53, SWI/SNF defects, epigenetic silencing, and impaired NIS transcription or localization form the malignant-cell basis of dedifferentiation. CAF metabolic reprogramming, TAM-associated inflammation, TGF-β, IL-6/CXCL8, hypoxia, and abnormal matrix and extracellular-vesicle-mediated communication can further stabilize poorly differentiated, invasive, and RAI-resistant states. Dedifferentiation is therefore better understood as a continuous process amplified and locked by the tumor ecosystem, not as a simple linear conversion from DTC to PDTC or ATC.

This view has therapeutic implications. Treatment should not focus only on transient restoration of differentiation genes after single-pathway inhibition. It should also ask whether NIS expression, membrane localization, functional iodine uptake, and microenvironmental dependency have been reversed. For RAI-refractory lesions that remain plastic, MAPK-directed redifferentiation, NIS-trafficking modulation, epigenetic intervention, and microenvironmental targeting may need to be combined according to the dominant functional defect. Ecosystem reprogramming will become clinically useful only when it restores a therapeutic ^131^I window and improves durable response. Extracellular vesicles may further propagate these signals between malignant, stromal, immune, and distant cells, although their direct role in NIS loss and RAI resistance remains to be established.

## Figures and Tables

**Table 2:** Experimentally supported tumor–microenvironment mechanisms in thyroid cancer dedifferentiation and progression.

Mechanism/Circuit	Main Source or Compartment	Experimental Support	Functional Consequence	Key References
CAF lactate-ZFP57/PKM2 axis	Cancer-associated fibroblasts and tumor cells	Co-culture and perturbation evidence links CAF-derived lactate to ZFP57/PKM2-dependent lineage suppression.	Supports dedifferentiation and may reduce thyroid-function programs.	[28]
TGF-β signaling	Tumor cells, CAFs and inflammatory stroma	BRAF-driven and stromal TGF-β pathways have been experimentally connected to NIS repression, EMT-like change and invasion.	Links oncogenic signaling to impaired iodine handling and invasive plasticity.	[38,40,41,43,63]
Inflammatory cytokine circuits	Tumor cells, immune cells and stromal cells	IL-6, CXCL8 and related cytokines are supported by cell-based and tissue-level studies, but direct redifferentiation endpoints vary.	Promotes inflammatory dedifferentiation niches, invasion and immune escape.	[26,42,43,63]
TAM enrichment and macrophage signaling	Tumor-associated macrophages	Advanced thyroid cancers show TAM enrichment; CXCL16 and macrophage-related studies support invasion-promoting effects, whereas direct lineage-restoration evidence remains limited.	Associated with invasion, immune suppression and poor outcome; dedifferentiation causality is partial.	[21,22,29,56,93]
Mast-cell IL-8-AKT-SLUG axis	Mast cells and tumor cells	Experimental studies show mast cells can induce EMT and stem-like features through IL-8-AKT-SLUG signaling.	Promotes EMT, stemness and invasion; not sufficient alone to define thyroid-lineage dedifferentiation.	[42,94]
Hypoxia/HIF-1α programs	Hypoxic tumor regions	Cell-based and mechanistic studies connect hypoxia/HIF-1α to altered metabolism, NIS regulation and RAI resistance.	Reinforces low-differentiation, metabolic adaptation and radioiodine resistance.	[39,83,95]
ECM stiffness/FAK/YAP-TAZ	Extracellular matrix, CAFs and tumor cells	Mechanotransduction studies and thyroid cancer models implicate matrix stiffness, FAK and YAP/TAZ in malignant progression.	Stabilizes invasion, plasticity and microenvironmental feedback; direct RAI-restoration evidence is still emerging.	[32,64,96,97]
Tumor-derived EV cargo	Tumor cells, CAFs, and immune cells	Thyroid cancer EV studies and functional co-culture assays support transfer of lncRNA, miRNA, or engineered cargo affecting macrophages, ECM remodeling, CD8+ T-cell exhaustion, or iodine avidity.	Propagates immunosuppression, matrix remodeling, migration, and potentially RAI resensitization; direct NIS-restoration evidence remains early.	[57,58,59,60,61]

Evidence levels are intentionally conservative: mechanisms are listed as dedifferentiation mechanisms only when thyroid-lineage or iodine-handling endpoints were assessed; otherwise they are described as invasion, EMT, immune or progression mechanisms.

**Table 3:** Mechanism-guided therapeutic strategies for redifferentiation and radioiodine resensitization.

Strategy	Main Target Level	Mechanistic Rationale	Evidence Status	Key Limitation	Key References
MAPK-pathway redifferentiation	BRAF, RAF or MEK signaling	Reduces MAPK output and can restore thyroid differentiation genes, NIS expression and RAI uptake.	Strongest translational evidence; mouse models and prospective clinical studies support restored uptake in selected tumors.	Responses depend on genotype, pathway rebound, lesion heterogeneity and whether uptake translates into absorbed dose.	[44,69,70,81,82,83,84,85,86,87,108]
BRAF/MEK plus feedback blockade	MAPK pathway with HER/RTK feedback	Counters adaptive HER3/RTK feedback that can limit RAF or MEK inhibitor effects.	Preclinical and early clinical evidence; useful as a combination concept.	Added toxicity and uncertain patient selection; needs pharmacodynamic endpoints.	[71,84]
PI3K-AKT-mTOR targeting	Survival and growth signaling	May cooperate with MAPK signaling to maintain low differentiation and RAI resistance.	Preclinical and organoid evidence supports combination logic.	Clinical redifferentiation evidence is weaker than for MAPK inhibition.	[72,73,103]
Epigenetic/chromatin intervention	SWI/SNF, HDAC, EZH2 or DNA methylation programs	May unlock silenced thyroid-lineage enhancers and improve reversibility of dedifferentiation.	Mechanistic evidence is growing; several approaches remain preclinical.	Risk of broad off-target effects; biomarkers of chromatin locking are not standardized.	[74,75,76,77,78,79,80,95,101,103,109,110]
NIS trafficking or membrane rescue	NIS glycosylation, localization and membrane retention	Addresses tumors with residual NIS expression but poor functional membrane transport.	Cell-based evidence supports restoring membrane localization and iodide uptake.	Requires functional uptake assays; NIS mRNA alone is insufficient for selection.	[4,33,95,99,100]
Hypoxia, ROS and metabolic stress targeting	HIF-1α, NOX4, PKM2, lactate and glycolysis	Targets stress programs that reinforce lineage loss, metabolic adaptation and RAI resistance.	Mechanistic and cell-based evidence; limited clinical validation.	May improve biology without restoring clinically meaningful iodine dose.	[28,39,43,64,77,78,79,80,111]
TME-directed redifferentiation support	CAF, TAM, TGF-β, IL-6/CXCL8 and ECM stiffness	Attempts to disrupt stromal and immune feedback that stabilizes dedifferentiated states.	Rationale supported by Table 2 mechanisms; direct redifferentiation evidence remains uneven.	Must show restoration of thyroid-lineage markers, NIS localization and RAI response, not only reduced invasion.	[26,28,29,32,41,42,43,56,57,58,59,60,61,62,63,64,65,66,67,68,94,96,97]
Endpoint-guided combination with RAI	Therapeutic workflow rather than single target	Combines pathway inhibition, NIS rescue and microenvironmental targeting with RAI only when uptake and dosimetry improve.	Conceptual but clinically relevant; aligns therapy with functional iodine-handling endpoints.	Requires standardized ^123^I/^124^I imaging, absorbed-dose estimates and post-RAI structural response.	[15,44,45,46,47,81,82,83,84,85,86,87,102]

The table emphasizes mechanism-guided use rather than routine combination therapy. The most clinically meaningful endpoint is restored lesion-level iodine uptake that produces an adequate absorbed dose and durable response after RAI.

**Table 4:** Clinically evaluated redifferentiation strategies followed by radioiodine treatment.

Molecular Context	Treatment Schedule	Uptake Restoration	Subsequent ^131^I	Clinical Response	Major Limitations
*BRAF^V600E^*-positive RAIR DTC	Dabrafenib + trametinib for 42 days; stimulated diagnostic scan on day 28	Abnormal uptake was 5% at baseline, 65% after treatment, and 95% on post-therapy scans among evaluable patients	5.5 GBq (150 mCi) ^131^I on day 35 after rhTSH	At 6 months: PR 38%, SD 52%, and PD 10%; selected patients could receive a second course [85]	Adverse events occurred in 96%; grade 3–4 events occurred in 7 patients; uptake and response remained heterogeneous
RAS-mutated RAIR DTC	Protocol-defined trametinib pretreatment followed by stimulated ^131^I in the MERAIODE phase II cohort	Restoration was limited and inconsistent; only a subset achieved sufficient uptake for treatment	Administered after post-treatment imaging and protocol-defined assessment	Among 10 evaluable patients: PR 20%, SD 70%, and PD 10% at 6 months [86]	Small single-arm cohort; modest redifferentiation activity; toxicity and patient selection remain concerns
BRAF/NRAS-mutated or other advanced RAIR thyroid cancer	Selumetinib 75 mg twice daily for 4 weeks with pre/post-treatment ^124^I dosimetry	New or increased uptake in 12/20 patients; 8/20 met the dosimetry threshold, including all 5 NRAS-mutant cases	^131^I was given during selumetinib treatment when lesion dosimetry met the protocol threshold	Among 8 treated patients: 5 PR and 3 SD; mean thyroglobulin reduction was 89% [44]	Small single-arm study; genotype and lesion heterogeneity affected eligibility and benefit
High-risk DTC after surgery, without established RAIR	Selumetinib 75 mg twice daily for approximately 5 weeks; RAI on treatment days 29–31	The trial tested complete remission rather than a dedicated uptake-restoration endpoint	100 mCi (3.7 GBq) ^131^I with rhTSH; study drug continued for 5 days	Complete remission at 18 months: 40% with selumetinib versus 38% with placebo; no significant benefit [102]	Adjuvant setting differs from RAIR disease; grade ≥ 3 treatment-related adverse events occurred in 16% [102]

Note: PR, partial response; SD, stable disease; PD, progressive disease; DTC, differentiated thyroid cancer; RAIR, radioactive iodine-refractory; RAI, radioactive iodine; rhTSH, recombinant human thyroid-stimulating hormone. Uptake restoration refers to lesion-level functional iodine uptake and should not be inferred from NIS transcript or protein expression alone.

## Data Availability

No new data were created or analyzed in this review. Data sharing is not applicable.

## Abbreviations

The following abbreviations are used in this manuscript:

AKT	protein kinase B
ATC	anaplastic thyroid carcinoma
BRAF	B-Raf proto-oncogene, serine/threonine kinase
CAF	cancer-associated fibroblast
CD8	cluster of differentiation 8
CXCL8	C-X-C motif chemokine ligand 8
CXCL16	C-X-C motif chemokine ligand 16
CXCR6	C-X-C motif chemokine receptor 6
DNMT1	DNA methyltransferase 1
DTC	differentiated thyroid cancer
ER	endoplasmic reticulum
ERK	extracellular signal-regulated kinase
EVs	extracellular vesicles
FAP	fibroblast activation protein
FN1	fibronectin 1
FOXE1	forkhead box E1
HER3	erb-b2 receptor tyrosine kinase 3
HIF-1α	hypoxia-inducible factor 1-alpha
IL-6	interleukin-6
IL-8	interleukin-8
MAPK	mitogen-activated protein kinase
MEK	mitogen-activated protein kinase kinase
MMPs	matrix metalloproteinases
mTOR	mechanistic target of rapamycin
NIS	sodium–iodide symporter
NKX2-1	NK2 homeobox 1
NOX4	NADPH oxidase 4
PAX8	paired box 8
PD	progressive disease
PD-1	programmed cell death protein 1
PD-L1	programmed death-ligand 1
PDTC	poorly differentiated thyroid carcinoma
PI3K	phosphoinositide 3-kinase
PKM2	pyruvate kinase M2
PR	partial response
PTC	papillary thyroid carcinoma
RAI	radioactive iodine
RAIR	radioactive iodine-refractory
RAS	rat sarcoma virus family
rhTSH	recombinant human thyroid-stimulating hormone
ROS	reactive oxygen species
SD	stable disease
SLC5A5	solute carrier family 5 member 5
SWI/SNF	switch/sucrose non-fermentable
TAM	tumor-associated macrophage
TAZ	transcriptional coactivator with PDZ-binding motif
TERT	telomerase reverse transcriptase
TG	thyroglobulin
TGF-β	transforming growth factor-beta
TP53	tumor protein p53
TPO	thyroid peroxidase
TSHR	thyroid-stimulating hormone receptor
UPR	unfolded protein response
VAV2	vav guanine nucleotide exchange factor 2
VEGF	vascular endothelial growth factor
WHO	World Health Organization
YAP	Yes-associated protein
ZFP57	zinc finger protein 57
ALDH	aldehyde dehydrogenase
ATF6	activating transcription factor 6
CCL21	C-C motif chemokine ligand 21
CCR7	C-C motif chemokine receptor 7
CD147	cluster of differentiation 147
CD31	cluster of differentiation 31
CD44	cluster of differentiation 44
CXCL12	C-X-C motif chemokine ligand 12
CXCR4	C-X-C motif chemokine receptor 4
CXCR7	C-X-C motif chemokine receptor 7
ECM	extracellular matrix
EMT	epithelial-mesenchymal transition
EZH2	enhancer of zeste homolog 2
FAK	focal adhesion kinase
IRE1	inositol-requiring enzyme 1
LYVE1	lymphatic vessel endothelial hyaluronan receptor 1
MMP2	matrix metalloproteinase 2
MMP9	matrix metalloproteinase 9
NF-kappaB	nuclear factor-kappa B
NRAS	neuroblastoma RAS viral oncogene homolog
NRG1	neuregulin 1
OCT4	octamer-binding transcription factor 4
PROX1	prospero homeobox 1
RTK	receptor tyrosine kinase
SLUG	snail family transcriptional repressor 2
SNAIL	snail family transcriptional repressor 1
SOX2	SRY-box transcription factor 2
TME	tumor microenvironment
TYMP	thymidine phosphorylase
VEGFR3	vascular endothelial growth factor receptor 3
ZEB1	zinc finger E-box binding homeobox 1
